# Gastrointestinal Symptoms of and Psychosocial Changes in Inflammatory Bowel Disease: A Nursing-Led Cross-Sectional Study of Patients in Clinical Remission

**DOI:** 10.3390/medicina56010045

**Published:** 2020-01-20

**Authors:** Rosellina Margherita Mancina, Raffaele Pagnotta, Caterina Pagliuso, Vincenzo Albi, Daniela Bruno, Pietro Garieri, Patrizia Doldo, Rocco Spagnuolo

**Affiliations:** 1 Department of Molecular and Clinical Medicine, The Sahlgrenska Academy at the University of Gothenburg, The Wallenberg Laboratory, Bruna Straket 16, SE-413 45 Gothenburg, Sweden; Rosellina.Mancina@wlab.gu.se; 2 University Medical Hospital “Mater Domini”, Viale Europa, 88100 Catanzaro, Italy; raffpagnotta@libero.it (R.P.); pagliusocaterina@gmail.com (C.P.); 3 School of Nursing, University “Magna Graecia”, Viale Europa, 88100 Catanzaro, Italy; vincealbi@hotmail.it (V.A.); soverato@unicz.it (D.B.); doldo@unicz.it (P.D.); 4 Department of Biomedical Sciences for Health, University of Milan, 20122 Milano, Italy; pietro.garieri@unimi.it; 5 Department of Clinical and Experimental Medicine, University “Magna Graecia”, Viale Europa, 88100 Catanzaro, Italy

**Keywords:** nurse–patient relationship, patient-centered care, questionnaire, quality of life, psychosocial nursing

## Abstract

*Background and Objectives*: Nursing management in Inflammatory Bowel Disease (IBD) is focused on global patient care. Starting from basic knowledge of diagnostic and therapeutic management, nurses can assess the impact of IBD on patients’ quality of life not only at the physical level, but also at the psychological, social, and emotional levels. The aim of this study was to evaluate the impact of gastrointestinal symptoms on psychosocial changes in IBD patients in remission through nursing-led Patient-Reported Outcomes. *Materials and Methods*: We performed a cross-sectional study of 109 IBD patients in clinical and endoscopic remission. Specialist nurses invited patients to complete questionnaires on gastrointestinal symptoms and quality of life through the Patient-Reported Outcomes Measurement Information System (PROMIS). *Results*: We found that the gastrointestinal symptoms that the patients reported had a significant impact on the analyzed aspects of health. More specifically, belly pain, diarrhea, and bloating were associated with depressive symptoms (*p* < 0.001), anxiety (*p* < 0.001), fatigue (*p* < 0.001), and sleep disturbances (*p* < 0.001). Moreover, these symptoms also significantly affected patients’ social dimension in terms of satisfaction with participation in social roles (*p* < 0.001, *p* < 0.05, and *p* < 0.001 for belly pain, diarrhea, and bloating, respectively) and physical functions (*p* < 0.001). The results were virtually the same in a multivariable analysis adjusted by age, gender, body mass index (BMI), and disease duration. *Conclusions*: Even during remission, gastrointestinal symptoms are the main factors that influence quality of life in IBD patients. This exploratory study highlights the need to adopt validated questionnaires in clinical practice, and demonstrates that PROMIS is a valid, objective, and standardized instrument that can help nursing staff to better define the consequences of the disease in a patient’s daily life.

## 1. Introduction

The 2nd Nursing–European Crohn Colitis Organization (N-ECCO) consensus on nursing management in patients with Inflammatory Bowel Disease (IBD) focuses on the role of nurses in global patient care, from basic knowledge of diagnostic and therapeutic management to the assessment of the disease’s impact on patients’ quality of life at the physical, psychological, social, and emotional levels [1]. IBD, which encompasses Crohn’s Disease (CD) and Ulcerative Colitis (UC), can have lifestyle impacts including self-imposed or professionally recommended dietary restrictions to control symptoms, long-term use of medication to control the disease [2], an immediate and lifelong psychological impact on daily activities, such as absences from school or work, and difficulties with meeting employment requirements [3]. Many people with IBD feel stigmatized, often because it is an intestinal disorder that others can perceive as dirty, or from the associated fatigue that others can misinterpret as laziness [4,5]. Some patients report feeling damaged due to physical changes associated with IBD or with its treatment. Furthermore, self-harm in relation to IBD onset may lead to a worse adaptation to the disease and a reduction in Health-Related Quality of Life (HRQoL) during remission and relapse [6,7]. Indeed, even in the remission phase, persistent disease-related problems, such as fatigue and sleep disorders, can be harmful to HRQoL. The most widespread and distressing symptoms that occur in people with IBD are pain, fatigue, sleep disorders, depression, and anxiety. These symptoms are more common during periods when the disease is active. However, they frequently persist during remission and continue to be considered a major disease-related concern. More specifically, 58.7–85.5% of IBD patients report pain [8,9,10,11,12]; 41–48% report fatigue [13]; 49–77% report sleep disorders [14]; and depressive symptoms and anxiety are experienced by 25–61% [12,15] and 31–44% [15,16], respectively. The reported prevalence of these symptoms among patients with IBD greatly varies, which is likely due to the different and non-standardized detection modalities. Objective tools for patient-reported outcome measurements (PROMs) are being developed to evaluate the impact of these concerns and the effectiveness of interventions on patients with IBD. PROMs are validated and standardized questionnaires that patients have completed regarding their perception of a health status or treatment without any interpretation by a caregiver [17]. The National Institutes of Health (NIH)’s Patient-Reported Outcomes Measurement Information System (PROMIS^®^) (http://www.nihpromis.gov) [18] is a set of publicly available and standardized Patient-Reported Outcomes (PROs) that cover physical appearance, mental health, and social health. In 2014, the GI PROMIS^®^ was developed, which comprises eight scales for gastrointestinal (GI) symptom domains [19]. A nested cross-sectional study on an internet-based cohort of patients with IBD from the Crohn’s and Colitis Foundation of America’s Partners showed that the GI-PROMIS domains were strongly associated with disease activity and Quality of Life indices [20]. Two recent studies on 5296 patients with IBD from the Crohn’s and Colitis Foundation of America’s Partners Cohort have used PROMIS to evaluate pain, fatigue, sleep disturbances, anxiety, and depression with the purpose of identifying symptom cluster membership among adults with IBD and examining associations between demographic and clinical factors (smoking status, time since diagnosis, medication, IBD type, and disease activity) [21,22]. The primary aim of this study is to evaluate the impact of gastrointestinal symptoms on psychosocial changes in IBD patients in clinical and endoscopic remission. A secondary aim is to quantitatively measure these characteristics through nursing-led Patient-Reported Outcomes.

## 2. Materials and Methods

### 2.1. Study Cohort

Consecutive outpatients from the IBD Unit of the University Hospital of Magna Graecia University of Catanzaro were recruited between March 2018 and September 2018. Patients were of either gender, greater than 18 years old, and had been diagnosed with IBD based on established clinical, endoscopic, radiological, and histological criteria. At the time of enrolment, IBD specialist nurses invited study participants to complete questionnaires on GI symptoms and quality of life through PROMIS^®^ as described below. Additionally, patients underwent a full evaluation of disease characteristics, including disease duration and location (Ileal, Ileo-Colonic, or Other Upper GI for CD or Proctitis, Proctosigmoiditis, Left-Side, or Pancolitis for UC), and previous surgery. Disease activity was evaluated by the Harvey Bradshaw index (HBI) [23] for Crohn’s Disease or by the Mayo Score (MS) [24] for Ulcerative Colitis. Information on medications (Mesalamine, Steroids, Thyopurine, and Anti TNF-alpha) was also collected. Only patients in remission with an HBI < 5 for CD or an MS < 2 for UC were included in the study.

### 2.2. Instruments

GI symptoms and quality of life in IBD patients were evaluated by GI-PROMIS^®^. The GI-PROMIS^®^ scales assess eight domains: gastroesophageal reflux (13 items), disrupted swallowing (7 items), diarrhea (5 items), nausea and vomiting (4 items), belly pain (6 items), and gas/bloating/flatulence (12 items) [19].

Satisfaction with participation in social roles and activities, pain interference, fatigue, sleep disturbances, depression, and anxiety were measured with PROMIS^®^. Eight-item PROMIS^®^ short forms were used to elicit responses from patients on each of the symptoms as occurring over the past 7 days. PROMIS^®^ items employ five-point Likert scales that range from 1 (not-at-all) to 5 (very much), with the exception of the items for pain interference, which include a score of 0 for no pain in addition to the five-point Likert scale. PROMIS^®^ measures are scored on a normalized *t*-score based on the general U.S. population, with 50 as the mean and 10-point standard deviation increments [25]. Furthermore, gastrointestinal and psychosocial symptom scores were dichotomized as present or absent based on a *t*-score cut-off of 50. More specifically, *t*-scores of 50 or higher indicate the presence of a clinically significant symptom, and *t*-scores lower than 50 indicate the absence of a clinically significant symptom. An exception is physical functions, in which the presence of alterations is given by a *t*-score value < 50 and the absence of alterations by a *t*-score value > 50 [25].

### 2.3. Statistical Analysis

Data were described using medians and quartile ranges or numbers and percentages as appropriate. Distributions of continuous variables across dichotomized psychosocial symptoms were compared by the Mann–Whitney non-parametric test for independent samples and by a linear regression analysis adjusted for age, gender, body mass index (BMI), and disease duration when appropriate. Traits were log-transformed before they were entered into the model. Categorical variables across dichotomized psychosocial symptoms were compared using Fisher’s exact test. A *p*-value < 0.05 was considered to be statistically significant.

### 2.4. Ethics

This study was approved by the local ethics committee of Magna Graecia University on 15 March, 2018 (protocol number 69). This study was conducted in compliance with the principles outlined in the Declaration of Helsinki. Informed written consent was obtained from each participating patient.

## 3. Results

### 3.1. Characteristics of the Study Group

Clinical and anthropometric characteristics of the study group are described in Table 1. Briefly, 109 patients with IBD (*n* = 78 (71%) UC; *n* = 32 (29%) CD) and a median age of 50 (42–59) years in clinical and endoscopic remission were included in the study. Of these, 69 (62%) were male. The median disease duration was 12 (0–34) years and only 13 patients (12%) had previously been subjected to surgery. Ninety-seven patients (84%) were treated with mesalamine, 9 patients (18%) were treated with Azathioprine, and 16 patients (14%) were treated with biological drugs.

Of the patients with CD, 15 (47%) had the terminal ileum as the disease location and a median HBI index of 2 (0–5). Of the patients with UC, 40 (51%) had an extension to the entire colon and a median MS of 1 (0–2).

### 3.2. Characteristics Of Gastrointestinal Symptoms and Psychosocial Alterations among IBD Patients

All patients completed the GI-PROMIS and PROMIS questionnaires as described in the methods section (Table 2). Of the 109 patients, most reported belly pain (*n* = 56, 51%), gas and bloating (*n* = 79, 72%), and diarrhea (*n* = 61, 56%) with a median *t*-score of 48 (33–58), 57 (47–62), and 52 (39–59), respectively. Forty-five patients (41%) reported symptoms associated with gastroesophageal reflux; 33 patients (30%) reported nausea and vomiting.

In the 7 days prior to the completion of the questionnaire, 76 patients (70%) experienced anxiety (median *t*-score of 54 (45–62)), 65 patients (60%) experienced fatigue (median *t*-score of 53 (42–60)), and 43 patients (39%) experienced depression (median *t*-score of 48 (38–56). Sleep disturbances were reported by 87 (79%) patients (median *t*-score of 53 (46–54)). About half of the patients (*n* = 46, 43%) reported alterations in their satisfaction with participation in social roles (median *t*-score of 53 (44–65)) and 62 patients (56%) reported that pain interfered with the normal performance of daily activities (median *t*-score of 55 (40–60)). Finally, 47 patients (43%) reported alterations in physical functions (median *t*-score of 50 (43–60)).

### 3.3. Gastrointestinal Symptoms Are Associated with Psychosocial Changes in IBD Patients

**Figure 1:**
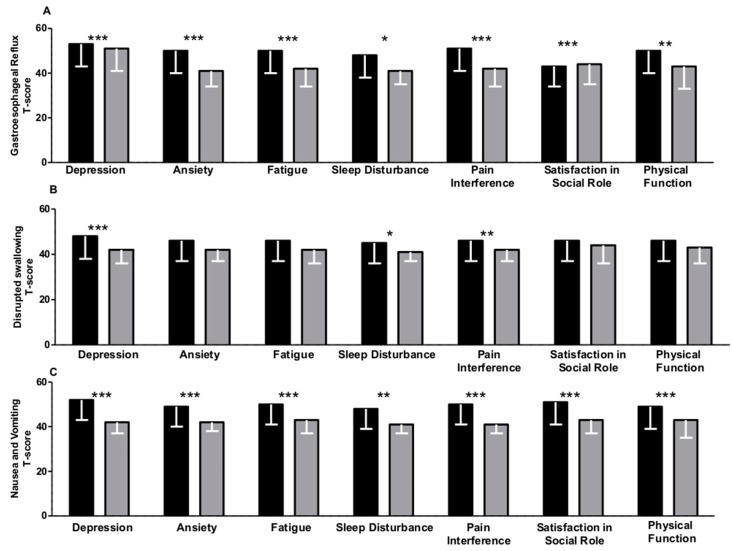
(**A**–**C**): Gastrointestinal symptoms in patients with Inflammatory Bowel Disease (IBD) as measured by GI-PROMIS^®^ scales across dichotomized psychosocial symptoms (Yes in Black/No in grey) (measured by PROMIS^®^) (see Methods section). A cut-off *t*-score of 50 was used to dichotomize symptoms. Data are expressed as the median and interquartile range. The overall *p*-value was calculated by the Mann–Whitney non-parametric test for independent samples. (*** *p* < 0.001; ** *p* < 0.005; * *p* < 0.05).

**Figure 2:**
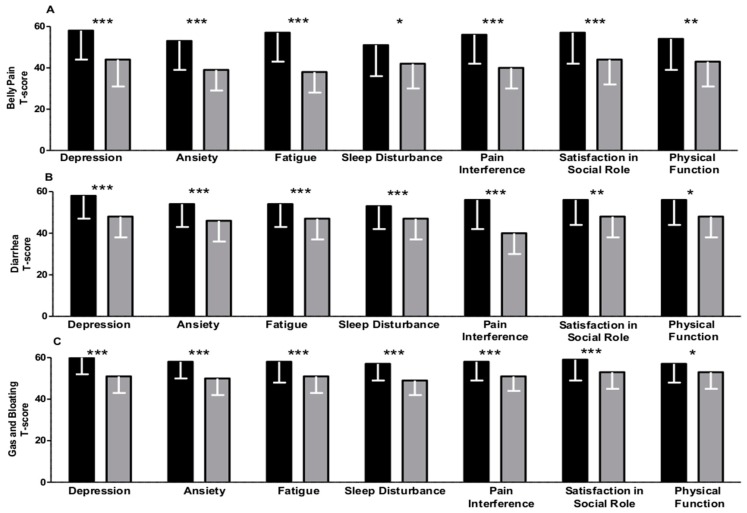
(**A**–**C**): Gastrointestinal symptoms in patients with IBD as measured by GI-PROMIS^®^ scales across dichotomized psychosocial symptoms (Yes in Black/No in grey) (measured by PROMIS^®^) (see Methods section). A cut-off *t*-score of 50 was used to dichotomize symptoms. Data are expressed as the median and interquartile range. The overall *p*-value was calculated by the Mann–Whitney non-parametric test for independent samples. (*** *p* < 0.001; ** *p* < 0.005; * *p* < 0.05).

As shown in Figure 1A–C and Figure 2A–C, the gastrointestinal symptoms that patients reported had a significant impact on the analyzed aspects of health. Belly pain (Figure 2A), diarrhea (Figure 2B), and bloating (Figure 2C) were associated with depressive symptoms (*p* < 0.001), anxiety (*p* < 0.001), fatigue (*p* < 0.001), and sleep disturbance (*p* < 0.001). Moreover, such symptoms also significantly affected the social dimension of these patients in terms of satisfaction with participation in social roles (*p* < 0.001, *p* < 0.05, and *p* < 0.001 for belly pain, diarrhea, and bloating, respectively) and physical functions (*p* < 0.001). In our study, these alterations were also found to be associated with symptoms in the high digestive tract, such as gastroesophageal reflux (Figure 1A) and nausea and vomiting (Figure 1C). No differences in relation to gender, smoking, type of IBD, or treatments were detected (see Supplementary Tables S1 and S2). The results were virtually the same in the multivariable linear regression model adjusted for age, gender, BMI, and disease duration (see Supplementary Table S3).

## 4. Discussion

This cross-sectional study, which was performed by dedicated nurses on IBD patients in remission, shows that gastrointestinal symptoms are the main determinants of psychosocial alterations in patients with IBD in remission as measured quantitatively by PROMIS. More specifically, we found that approximately half of the patients (51%) reported belly pain, 56% reported diarrhea, and 72% reported gas and bloating. These symptoms were associated with psychological alterations, including anxiety, depression, fatigue, and sleep disturbances, and also affected the social dimension in terms of the ability to participate in social roles and physical functions. In a previous retrospective study with a cohort of 5296 IBD patients, Conley et al. [21], in a nursing management context, showed that ~38% of these patients reported a symptom cluster as measured by a latent class analysis and characterized by a high probability of experiencing such psychological symptoms as those described in our study, but not those of the social dimension. Furthermore, in this study, in the subgroup analysis, 19% of patients were in remission with depression, 30% of patients were in remission with anxiety, 25% of patients were in remission with asthenia, and ~26% of patients were in remission with a sleep disorder. These data differ considerably in our study, in which—using PROMIS—we detected depression in ~40% (43/109), anxiety in ~70% (76/109), asthenia in 60% (65/109), and a sleep disorder in 80% (87/109) of our IBD patients in clinical remission. To our knowledge, only two previous studies have used GI PROMIS^®^ questionnaires to identify gastrointestinal symptoms in patients with IBD [19,20]. In the study by Kochar et al. the GI PROMIS was used in a cohort of 2378 CD and 1455 UC patients with the result that the domains closely correlated with other quality-of-life indicators. To date, no studies have used GI PROMIS domains in correlation with psychosocial symptoms with the aim of identifying an association between alterations in psychosocial symptoms and disease activity and quality of life. In our cross-sectional study, for the first time, we submitted a GI PROMIS^®^ questionnaire to a small selected cohort of IBD patients in remission and we have shown that gastrointestinal symptoms were associated with psychosocial alterations. Of note, these changes were not associated with the type of IBD, differences in treatments, disease duration, or previous surgical procedures. Moreover, using a multivariable analysis, we found that these associations were independent of age, gender, BMI, and duration of disease. A sensitivity analysis separated by gender would help to confirm these results. However, due to the relatively low number of patients in the study cohort, further stratification of individuals based on gender or disease localization is likely to fail to detect differences due to power issues. Interestingly, our data show that, even during remission, gastrointestinal symptoms are the main variables that influence quality of life in IBD patients. Possible reasons for the persistence of symptoms may include sub-clinical inflammation and irritable bowel syndrome [26,27,28]. Furthermore, as a secondary aim, our study highlights the strategic importance of the use of PROMIS as an objective and standardized tool to help nurses evaluate the effect of symptoms on IBD patients’ every-day life. In fact, while the role of nursing care in the treatment of IBD is well established, there is currently no appropriate definition of the specific outcomes that are sensitive to the quantification of the nursing intervention.

## 5. Conclusions

Together with previous results, our small exploratory study highlights that, in a group of IBD patients in clinical remission, gastrointestinal symptoms are the main cause of alterations in physical and social dimensions. Hence, there arises a need to adopt validated questionnaires such as PROMIS^®^ not only in research studies focused on patient-centered outcomes, but also in clinical practice where both medical and nursing staff can use these instruments to better define the consequences of the symptoms on a patient’s daily life, particularly where, according to arbitrary measures, the patient seems well. Recognition of such alterations could allow health staff to identify the real needs of these patients. Overall, therefore, the role of nursing will be to identify the most suitable treatment approach in a holistic manner. Further studies will be needed to determine prospectively which personalized interventions need to be applied and to validate these questionnaires with a larger sample size.

## Figures and Tables

**Table 1:** Characteristics of the study cohort.

*N*	109
**Demographic and Anthropometric**	
Age (years)	50 (42–59)
Male gender, *n* (%)	69 (62)
BMI (Kg/m^2^)	25 (23–27)
Smoke, *n* (%)	14 (13)
**Disease characteristics**	
Crohn’s disease, *n* (%)	32 (29)
Ulcerative Colitis, *n* (%)	78 (71)
Disease duration (years)	12 (0–34)
CD (Harvey Bradshaw index)	2 (0–5)
UC (full Mayo Score)	1 (0–2)
Surgery, *n* (%)	13 (12)
**CD disease location, *n* (%)** **†**	
Ileal	15 (47)
Ileo-Colonic	13 (41)
Other Upper GI	4 (12)
**UC disease location, *n* (%) ‡**	
Proctitis	5 (6)
Proctosigmoiditis	10 (13)
Left-side	20 (26)
Pancolitis	40 (51)
**Medications, *n* (%)**	
Mesalamine	97 (84)
Steroids	10 (9)
Thyopurine	9 (8)
Biological	16 (14)

Continuous variables are expressed as the median and interquartile range. Categorical variables are expressed as the number and proportion. † Data refer to 32 individuals with Crohn’s Disease (CD), ‡ data refer to 78 individuals with Ulcerative Colitis (UC). Abbreviations: BMI: Body Mass Index.

**Table 2:** Gastrointestinal Patient-Reported Outcome Measurement Information System (GI-PROMIS) and Psychosocial Patient-Reported Outcome Measurement Information System (PROMIS) domains in Inflammatory Bowel Disease (IBD).

*N* = 109	Yes	Median *t*-Score ± SD
**GI-PROMIS**		
Belly pain, *n* (%)	56 (51)	48 (33–58)
Gas and Bloating, *n* (%)	79 (72)	57 (47–62)
Diarrhea, *n* (%)	61 (56)	52 (39–59)
Gastroesophageal Reflux, *n* (%)	45 (41)	47 (36–52)
Disrupted Swallowing, *n* (%)	20 (18)	46 (40–49)
Nausea and Vomiting, *n* (%)	33 (30)	47 (40–55)
**PROMIS**		
Depression, *n* (%)	43 (39)	48 (38–56)
Anxiety, *n* (%)	76 (70)	54 (45–62
Fatigue, *n* (%)	65 (60)	53 (42–60)
Sleep Disturbance, *n* (%)	87 (79)	53 (46–54)
Pain interference, *n* (%)	62 (56)	55 (40–60)
Satisfaction with participation in social roles, *n* (%)	46 (43)	53 44–65)
Physical Function, *n* (%)	47 (43)	50 (43–60)

Continuous variables are expressed as the median and interquartile range. Categorical variables are expressed as the number and proportion. The gastrointestinal (GI) and psychosocial PROMIS domains are scored on a *t*-score metric with a mean of 50 and a standard deviation (SD) of 10 in the U.S. general population. Gastrointestinal and psychosocial symptom scores were dichotomized for the use of categorical variables.

## Supplementary Materials

The following are available online at https://www.mdpi.com/1010-660X/56/1/45/s1: Table S1: Categorical variables dichotomized across gastrointestinal symptoms; Table S2: Categorical variables dichotomized across psychosocial symptoms; Table S3: Multivariable linear regression model adjusted for age, gender, BMI, and disease duration.
