# Supplementary material for: Gastrointestinal Symptoms of and Psychosocial Changes in Inflammatory Bowel Disease: A Nursing-Led Cross-Sectional Study of Patients in Clinical Remission
Source: Medicina (Kaunas). 2020 Jan 20;56(1):45. doi: 10.3390/medicina56010045 (PMC7022245; doi:10.3390/medicina56010045)
Supplement: Supplementary file 1 [file medicina-56-00045-s001.zip › supplementary files/Supplementary Table 2 .docx]

| **Supplementary Table 2**  **Categorical variables dichotomized across psychosocial symptoms** | | | | | | | | | | | | | | | | | | | | | |
| --- | --- | --- | --- | --- | --- | --- | --- | --- | --- | --- | --- | --- | --- | --- | --- | --- | --- | --- | --- | --- | --- |
|  | Depression | | *p* | Satisfaction to partecipate in social roles | | *p* | Anxiety | | *p* | Fatigue | | *p* | Pain interference | | *p* | Sleep disturbance | | *p* | FF | | *p* |
|  | Y | N |  | Y | N |  | Y | N |  | Y | N |  | Y | N |  | Y | N |  | Y | N |  |
| GENDER | | | | | | | | | | | | | | | | | | | | | |
| M | 26 | 43 | 0.6 | 25 | 44 | 0.1 | 44 | 25 | 0.08 | 38 | 31 | 0.2 | 36 | 33 | 0.2 | 54 | 15 | 0.8 | 8 | 61 | 0.06 |
| F | 17 | 23 |  | 21 | 19 |  | 32 | 8 |  | 27 | 13 |  | 26 | 14 |  | 33 | 7 |  | 11 | 29 |  |
| SMOKING | | | | | | | | | | | | | | | | | | | | | |
| Y | 8 | 6 | 0.1 | 5 | 9 | 0.7 | 11 | 3 | 0.5 | 9 | 5 | 0.7 | 7 | 7 | 0.5 | 13 | 1 | 0.2 | 1 | 13 | 0.4 |
| N | 35 | 60 |  | 41 | 54 |  | 65 | 30 |  | 56 | 39 |  | 55 | 40 |  | 74 | 21 |  | 18 | 77 |  |
| IBD | | | | | | | | | | | | | | | | | | | | | |
| CD | 17 | 15 | 0.1 | 15 | 17 | 0.1 | 7 | 25 | 0.06 | 22 | 10 | 0.08 | 25 | 7 | 0.05 | 27 | 5 | 0.1 | 5 | 27 | 0.2 |
| UC | 26 | 51 |  | 30 | 47 |  | 28 | 50 |  | 42 | 35 |  | 42 | 35 |  | 60 | 17 |  | 16 | 61 |  |
| SURGERY | | | | | | | | | | | | | | | | | | | | | |
| Y | 4 | 9 | 0.5 | 6 | 7 | 0.7 | 8 | 5 | 0.5 | 7 | 6 | 0.7 | 8 | 5 | 0.7 | 10 | 3 | 0.7 | 4 | 9 | 0.2 |
| N | 38 | 58 |  | 43 | 53 |  | 64 | 32 |  | 55 | 41 |  | 56 | 40 |  | 73 | 23 |  | 19 | 77 |  |
| MESALAZINE | | | | | | | | | | | | | | | | | | | | | |
| Y | 38 | 59 | 1.0 | 42 | 55 | 0.5 | 67 | 30 | 0.7 | 55 | 42 | 0.1 | 55 | 42 | 0.7 | 76 | 21 | 0.2 | 15 | 82 | 0.07 |
| N | 5 | 7 |  | 5 | 7 |  | 8 | 4 |  | 8 | 4 |  | 7 | 5 |  | 12 | 0 |  | 5 | 7 |  |
| STEROIDS | | | | | | | | | | | | | | | | | | | | | |
| Y | 4 | 6 | 1.0 | 6 | 4 | 0.1 | 6 | 4 | 0.4 | 6 | 4 | 1.0 | 6 | 4 | 0.7 | 6 | 4 | 0.2 | 3 | 7 | 0.1 |
| N | 39 | 60 |  | 40 | 59 |  | 65 | 34 |  | 56 | 43 |  | 52 | 47 |  | 73 | 26 |  | 21 | 78 |  |
| AZATYOPRINE | | | | | | | | | | | | | | | | | | | | | |
| Y | 0 | 9 | 0.05 | 2 | 7 | 0.2 | 4 | 5 | 0.1 | 4 | 5 | 0.4 | 5 | 4 | 1.00 | 5 | 4 | 0.1 | 0 | 9 | 0.3 |
| N | 42 | 58 |  | 44 | 56 |  | 72 | 28 |  | 60 | 40 |  | 56 | 44 |  | 82 | 18 |  | 19 | 81 |  |
| BIOLOGICS | | | | | | | | | | | | | | | | | | | | | |
| Y | 11 | 5 | 0.05 | 10 | 6 | 0.1 | 12 | 4 | 0.7 | 10 | 6 | 1.0 | 8 | 8 | 0.5 | 13 | 3 | 1.0 | 2 | 14 | 0.7 |
| N | 33 | 60 |  | 37 | 56 |  | 64 | 29 |  | 54 | 39 |  | 53 | 40 |  | 73 | 20 |  | 20 | 73 |  |
| Categorical variables dichotomized across psychosocial symptoms were compared using the Fisher-Exact test. *p-*value < 0.05 was considered significant. Abbreviations: IBD: Inflammatory Bowel Disease; FF: Physical Function | | | | | | | | | | | | | | | | | | | | | |
